# Basic Life-Support Learning in Undergraduate Students of Sports Sciences: Efficacy of 150 Minutes of Training and Retention after Eight Months

**DOI:** 10.3390/ijerph16234771

**Published:** 2019-11-28

**Authors:** Silvia Aranda-García, Ernesto Herrera-Pedroviejo, Cristian Abelairas-Gómez

**Affiliations:** 1 GRAFIS Research group, Institut Nacional d’Educació Física de Catalunya (INEFC), Universitat de Barcelona (UB), 08038 Barcelona, Spain; 2 Health and Applied Sciences Department, Institut Nacional d’Educació Física de Catalunya (INEFC), Universitat de Barcelona (UB), 08038 Barcelona, Spain; 3 Physiotherapy Department, Faculty of Medicine and Health Sciences, Universitat Internacional de Catalunya, 08195 Sant Cugat del Vallès, Spain; 4 Blanquerna School of Health Sciences-Ramon Llull University, 08025 Barcelona, Spain; 5 CLINURSID Research Group, Universidade de Santiago de Compostela, 15782 Santiago de Compostela, Spain; cristianabelairasgomez@gmail.com; 6 Faculty of Education Sciences, Universidade de Santiago de Compostela, 15782 Santiago de Compostela, Spain; 7 Institute of Health Research of Santiago (IDIS), Santiago de Compostela, 15706 Santiago de Compostela, Spain

**Keywords:** basic cardiac life support, cardiopulmonary resuscitation, retention, exercise, sports, feedback, follow-up studies, simulation training

## Abstract

Several professional groups, which are not health professionals, are more likely to witness situations requiring basic life support (BLS) due to the nature of their job. The aim of this study was to assess BLS learning after 150 min of training in undergraduate students of sports science and their retention after eight months. Participants trained on BLS (150-min session: 30 theory, 120 practice). After training (T1) and after 8 months (T2), we evaluated their performance of the BLS sequence and two minutes of cardiopulmonary resuscitation (CPR). At T1, the 23 participants presented a mean score of 72.5 ± 21.0% in the quality of the CPRs (compressions: 78.6 ± 25.9%, ventilation: 69.9 ± 30.1%). More than 90% of the participants acted correctly in each step of the BLS sequence. At T2, although the overall quality of the CPR performed did not decrease, significant decreases were observed for: correct hand position (T1: 98.2 ± 8.8, T2: 77.2 ± 39.7%), compression depth (T1: 51.4 ± 7.9, T2: 56.0 ± 5.7 mm), and compression rate. They worsened opening the airway and checking for breathing. In conclusions, participants learned BLS and good-quality CPR after the 150-min training session. At eight months they had good retention of the BLS sequence and CPR skills. Training on airway management and the position of the hands during CPR should be reinforced.

## 1. Introduction

A person with out-of-hospital cardiac arrest (OHCA) does not always receive cardiopulmonary resuscitation (CPR) [1] presumably due to a lack of competence of the bystanders witnessing the emergency [2]. For this reason, the European Resuscitation Council (ERC) recommends that the general population should be taught basic life support (BLS) [2]. There are several professional groups that should know BLS because they are more likely to be present when OHCAs occur and their first-aid actions could be decisive in such situations. These groups include exercise professionals, who work in environments involving a large number of people (e.g., pavilions, sports stadiums and sport institutes) and/or people with cardiovascular risk factors (e.g., personal training sessions for exercise prescriptions). Most Spanish regional laws that regulate sports professions highlight the need for competence in CPR [3,4]. Thus, exercise professionals should be trained in BLS, for example, during their studies.

The learning of BLS can be determined by factors related to the student (i.e., educational level or age) [5] or to the type of training (i.e., the use of feedback devices, simulated environments, teacher-student ratios or the duration of the training) [2,6].

Although there is no ideal method for learning BLS [7,8], there are certain aspects that have been shown to be effective. For example, feedback devices are especially useful in the learning of CPR skills as they correct the technique in real time and improve its learning [9] by correcting parameters such as the compression rate, depth, full recoil and hand positions [2]. Specific simulated scenarios based on the learner’s workplace [10] have also been shown to be effective, ensuring safe practice and providing an appropriate and specific context for learning [11]. The simulated training in hospital settings for health professionals reported in [10] could be adapted to each profession (i.e., sports environments for exercise professionals) to provide appropriate training.

In addition to learning BLS skills, it is also important to retain them over time. The ability to perform BLS correctly decreases with the time elapsed since training [12,13,14,15,16,17]. In health professionals, it is recommended that there should be no more than 3–6 months between training and refresher sessions [10] and that refresher training should ideally occur monthly [12]. Although there is some information that also indicates that refresher training should not exceed 3–6 months in lay people [2,13], more studies are needed in this population before giving a clear recommendation about the frequency of retraining.

We hypothesized that after a training session, people from the field of sports sciences can perform good BLS, including quality CPR. They can retain those abilities as well or better than other lay people or people from the field of health. Therefore, the objectives of this study were to: (1) describe the learning of the BLS sequence and the quality of the CPR performed after 150 min of training in undergraduate students of sports science, and (2) analyse the retention of the BLS sequence and CPR skills 8 months after the training.

## 2. Materials and Methods

### 2.1. Participants

**Figure 1:**
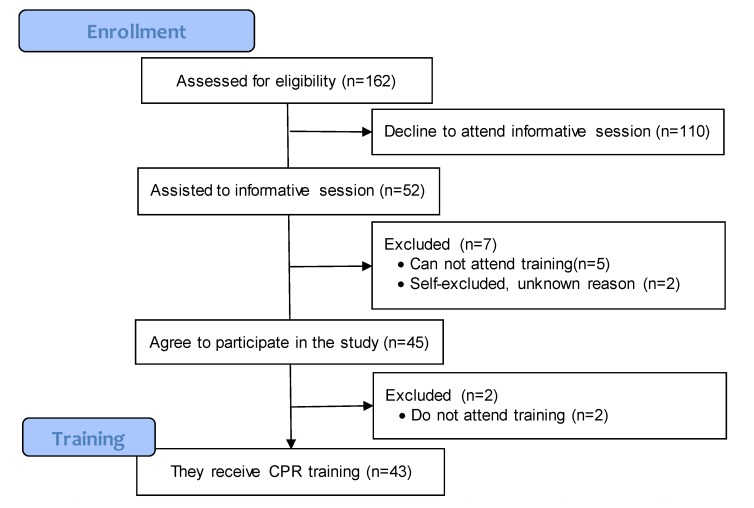
Flow diagram of participant’s recruitment.

We contacted 162 undergraduate students of Physical Activity and Sports Science degree from the public university of Barcelona (INEFC-Barcelona). Fifty-two of them attended an informative meeting where the study was explained, 45 accepted voluntarily to participate in the study, and 43 received the training (Figure 1). The inclusion criteria were to be a third-grade student (where the first aid subject is taught), not having previously received BLS training, have availability to attend training and not have physical impediments to perform CPR. Participants who could not attend training or the follow up were excluded. All participants were informed about the aim of the research, the study design, the confidentiality statement, and that their participation was voluntary and they could withdraw if they wished.

### 2.2. Study Design

**Figure 2:**
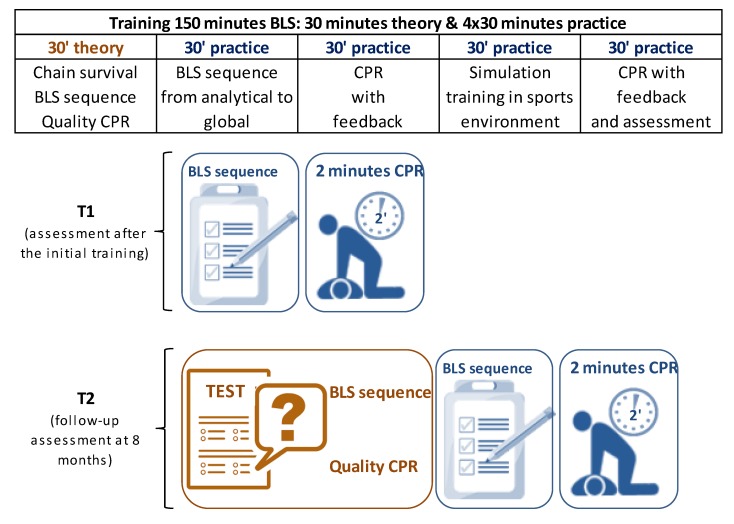
Study design schema. Assessments at T1 (after initial training) and T2 (follow-up at eight months). BLS: basic life support, CPR: cardiopulmonary resuscitation.

A quasi-experimental study without a control group was performed, with a follow-up at eight months. Participants were trained in BLS and were assessed after this training (T1). We encourage to all participants to attend the follow-up assessment at eight months (Figure 2).

### 2.3. Training

To ensure uniform training, all the trainers were emergency and teaching professionals who were accredited by the *Consell Català de Ressuscitació* and approved by the *Consejo Español de Reanimación Cardiopulmonar* and the ERC. Each group contained a maximum of eight students for one teacher and had three practice manikins (QCPR, Laerdal Medical^®^).

The training lasted 150 min and consisted of 30 min of theory and 120 min of practice (Figure 2). The theory part included concepts related to BLS (survival chain, the BLS sequence and CPR), emphasising the key aspects associated with high-quality CPR following the recommendations of the ERC-2015 [2]. The practice part of the training was divided into four 30-min blocks that involved: (a) learning the BLS sequence using a methodology from the analytical to the global, in which the teacher first performed the demonstration and then the students practised it; (b) practising CPR (standard sequence of 30 compressions: 2 ventilations) with a biofeedback system and real-time correction of the chest compressions, depth and ratio (SkillGuide, Laerdal Medical); (c) practical cases in a simulated scenario of a sports event (i.e., cardiac arrest of a match spectator); and (d) combined practice with a biofeedback system involving a BLS case and two consecutive minutes of CPR.

### 2.4. Procedure, Assessments and Variables

Before the training, we registered participants’ personal data (age, sex, etc.). After training (T1) and at eight months (T2), we evaluated the execution of the BLS sequence and two consecutive minutes of CPR (30:2) by the participants (Figure 2). Furthermore, the participants were asked to answer three questions related to CPR at T2: position of hands, proportion of compressions–ventilations, chest compression depth and rate (Table 1). The BLS sequence was evaluated in a simulated OHCA scenario. Participants had to recognise the incident of the cardiac arrest and follow the BLS sequence steps and perform two minutes of CPR. The scenario for each participant involved an unconscious victim who was not breathing in a sporting environment.

We assessed the steps of the BLS sequence qualitatively using an observation checklist (Table 1). The BLS sequence consisted of: confirming the safety of the environment, checking for consciousness, opening the airway, checking for breathing, calling 112 (emergency phone) for help and starting CPR. We obtained the variables related to CPR skills (Table 1) with the Resusci Anne Wireless SkillReporter software (v. 2.0.0.14) (Laerdal Medical^®^). Finally, we asked the participants for their subjective perception regarding their level of competence in performing CPR and their self-reported confidence in performing CPR in a real situation of an OHCA. We measured these variables with a 10-cm visual analogue scale.

### 2.5. Ethical Considerations

The study was approved by the Clinical Research Ethics Committee of Sports Administration of Catalonia (reference number 09/2016/CEICEGC). All the students provided written informed consent before their participation.

### 2.6. Data Analysis

Variables were expressed as mean (standard deviation) or absolute frequencies (relative frequencies) as appropriate. The Shapiro-Wilk test was used to check the normal distribution of the continuous variables (CPR variables). Paired samples *T*-test or Wilcoxon Signed Rank test were used to compare CPR variables between T1 (after initial training) and T2 (after 8 months); the *T*-test in the case of parametric variables and Wilcoxon Signed Rank test when data did not follow a normal distribution. McNemar’s test was carried out to compare dichotomous variables over time; those variables were the steps of BLS sequence, which were categorized as yes/no (performed/not performed). All statistical analyses were performed using SPSS for Windows (v. 21.0), with the significance level set at *p* < 0.05.

## 3. Results

Of the 43 participants who received the training, 24 attended the follow-up (2 did not want to attend the follow-up, and 17 were self-excluded for unknown reasons). Finally, 23 participants were analyzed because one of them was excluded due to an error in the data. Of those 23 participants evaluated, 39% were female and all were aged between 21 and 28 years.

### 3.1. Basic Life Support Learning

After the training (T1), almost 100% of the participants performed the BLS sequence perfectly. All of them adequately assessed the safety of the environment, managed the airway and started CPR. Of the 23 participants, 19 (82.6%) performed all the steps of the BLS sequence correctly, 21 (91.3%) checked for consciousness and 22 (95.7%) checked for breathing and called 112 for help.

At T1, the participants presented a mean score of 72.5% (±21.0%) for their global CPR performance, 78.6% (±25.9%) for the chest compressions they performed, and 69.9% (±30.1%) for the ventilations they performed. Chest compressions were performed with a mean depth of 51.4 mm (±7.9 mm) and a mean rate of 115.3 compressions/min (±11.5 compressions/min), with 98.2% (±8.8%) of the compressions performed with the correct hand positions. The ventilations were performed with a mean volume of 663.7 mL (±319.0 mL) and a mean hands-off time of 7.5 s (±2.1 s). Table 2 presents all the descriptive data regarding the CPR skills.

The descriptive analysis of the participants who obtained a CPR performance score of <70% (*n* = 9; 39%) revealed a mean score of 52.1% (±6.1%, range: 21–69%) in their total CPR performance, 54.7% (±9.0 %) for the chest compressions they performed (67.4% (±12.6%) of the compressions were too shallow) and 73.3% (±11.6%) for the ventilations they performed. Table 3 shows all the descriptive data of the variables related to the global CPR performance of these participants at T1.

### 3.2. Basic Life-Support Retention

**Figure 3:**
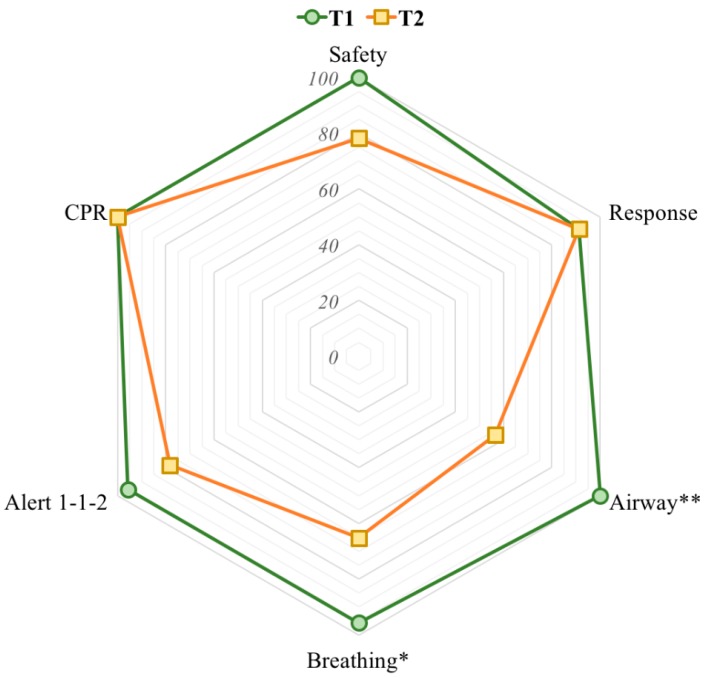
Percentage of participants who correctly performed the BLS sequence at T1 (green) and T2 (yellow). * *p* < 0.05, ** *p* < 0.01.

When comparing T1 and T2, a smaller proportion of the participants performed each step of the BLS sequence, except for checking for consciousness and starting CPR, at T2. The differences were statistically significant for airway opening and checking for breathing between T1 and T2 (*p* = 0.002 and *p* = 0.016, respectively). Figure 3 shows the percentages of the participants who correctly performed each step of the BLS sequence after training and at follow-up. At T2, only eight (34.8%) participants performed all the steps of the BLS sequence correctly (*p* = 0.007).

There were no significant differences in the global CPR performance, compressions and ventilations between T1 and T2 (Table 2). However, both the global quality of compressions and ventilations are the sum of other different variables. For compressions, quality is determined by the depth of the compression, the rate and the position of the hands. The mean chest compression depth increased significantly eight months after training (*p* = 0.007), with the percentage of compressions performed at an adequate depth increasing and those that were too shallow decreasing (*p* = 0.010 in both cases). Additionally, in participants with a global CPR performance score greater than 70%, we found no significant changes between T1 (*n* = 14; 60.9%) and T2 (*n* = 13; 56.5%). For ventilations, quality depends on the ventilation volume. There were no differences found in the quality of the ventilations between T1 and T2.

At T2, 10 (43.5%) participants correctly answered the question about the placement of the hands for chest compression, nine (39.1%) about the chest compression depth and 13 (56.5%) about the chest compression rate. Only three (13.0%) participants correctly answered all three questions. Additionally, the self-reported level of competence and confidence in performing CPR in real situations had a mean score of 67.9% (±0.8) and 64.6% (±1.6), respectively, at T2.

## 4. Discussion

After the 150-min BLS training, our participants showed good CPR skills when attending to an OHCA in a simulated sports scenario. Furthermore, most of the participants correctly performed each of the steps of the BLS sequence, as reported in other studies using a shorter [18,19] or longer [20] training session than that used in our study. Our study participants achieved a mean global CPR performance score of greater than 70%, which is considered optimal [21]. These results are in accordance with those of a previous study evaluating university students in the health field, which reported that 96% of their study participants achieved global CPR performance scores >70% [22]. However, there are also some studies that have reported poor CPR performance scores (mean scores <50% [23], or 69% of participants <70% CPR [19]). Although to the best of our knowledge there are no previous studies assessing university students in sports sciences for us to compare our results to, we did compare our findings with those evaluating lay participants and found that our study participants obtained similar [24] or even better performance scores [5]. Specifically, a study involving 90 university students in the field of education reported CPR performance scores that were very similar to ours (80% of chest compressions and 37% of ventilations performed correctly) [24], while another study assessing 80 lay participants after a half-day training session reported a CPR performance score of 41% [5], which was much lower than that found in our study.

The high quality of the CPR performed by our study participants was due to the better chest compressions (78.6%) performed compared to the ventilations (69.9%). In general, our participants obtained satisfactory results in all the key aspects of performing chest compressions, similar to that reported by previous studies. Nearly all the compressions were performed with the correct hand positions (98.2%) [25], more than two-thirds of the chest compressions had a depth of 50–60 mm [26,27], more than half of the chest compressions were performed at a rate of 100–120 compressions/min [28], and each cycle of 2 ventilations had a mean time of 7.5 s, which is below the recommended maximum of 10 s [2]. It should be noted that the low scores of the nine participants who had global CPR performance scores <70% after their training were due to performing chest compressions that were too shallow. Performing compressions below the minimum recommended depth has previously been described to be a common error [20].

Regarding the ventilations, although the mean volume (663.7 mL) was not very far from the maximum recommended threshold of 600 mL, only 38.2% of the ventilations performed were within the desired range of volumes. Given that ventilations are more difficult to perform than chest compressions for lay people [29] and that the ability to perform them decreases earlier [13], it was not surprising that our participants from the field of sports sciences did not present excellent results for performing ventilations.

Although a lower percentage of the participants performed each step of the BLS sequence correctly (except starting CPR) eight months after their training, we only found significant differences for the steps that involved opening the airway and checking for breathing between T1 and T2. A recent study reported similar results [18], observing differences for confirming the safety of the environment and checking for consciousness eight weeks after training compared to just after the training. Although opening the airway and checking for breathing are related to the airway and could be considered important for identifying an OHCA, retaining knowledge on these BLS steps is likely to decrease in the long term, such as after eight months [10,30].

Our finding that CPR skills did not significantly worsen eight months after training is remarkable. This was corroborated by no significant changes in global CPR performance, as well as in the chest compressions and ventilations performed. Therefore, our results indicate that frequent BLS retraining, every 3–6 months as recommended for professionals in the health field [10,30], might not be necessary. Likewise, our results provide evidence called for in previous publications concerning retention of CPR skills beyond six months [5]. To our knowledge, this has not been described before for exercise professionals.

In addition to the factors previously described (i.e., the type of training, age and educational level) [5,30], the capacity to retain CPR skills could also be related to greater motor skills. Greater motor skills can facilitate the learning and retention of a new motor skill due to the transfer principle, that is, the ability to learn a skill when one has already been acquired [31]. This same principle also explains the capacity to retain the skill learned over time [32]. The curriculum of the degree our participants were studying includes a large amount of content and practice. This, together with the practice of extracurricular sporting activities [33], is likely to increase the motor skills of our participants, thereby enhancing their ability to learn and retain new skills such as performing CPR.

There was a significant decrease in the correct hand positions used for chest compressions eight months after training, which resulted in low-quality CPR. Moreover, less than half of the participants correctly answered the theoretical question about the exact point of compression. Since a previous study also described that this aspect was likely to be forgotten four months after BLS training [34], special emphasis should be placed on ensuring correct hand placement in both the initial and refresher training sessions.

The participants were also asked to report their confidence about performing CPR in a real situation and their level of competence eight months after training. Previous studies have described that confidence increases significantly after BLS training [18,20], but decreases after periods of no refresher training, such as in our study, with values below 70% [18]. Low confidence and/or competence could affect the decision to provide first aid in an emergency, which justifies the need for so-called rolling refreshers (short and periodic training) [6].

This study was not free from limitations. It used a simulated environment with manikins; thus, our results cannot be extrapolated to real situations involving more stress. Furthermore, the study design, an eight-month longitudinal study, affected the size of the sample. Additionally, our participants were recruited from only one university and voluntarily, therefore, there could be some bias related to economic, social, gender or cultural aspects.

## 5. Conclusions

University students in sports sciences were able to effectively attend to a simulated OHCA after a 150-min training session. Although the retention of CPR skills 8 months after the training was good, airway management and breathing assessment, as well as correct hand positioning during CPR, had significantly worsened. This should be taken into account in the initial training and refresher sessions of BLS in people with similar profiles to those of our study participants.

## Figures and Tables

**Table 1:** List of variables resulting from the CPR test, BLS checklist, questionnaire, and self-perception.

**CPR**	**BLS Checklist**
Global CPR performance (%)	Checking for consciousness
Compression performance (%)	Opening the airway
Ventilation performance (%)	Checking for breathing
Compression depth (mm)	Calling 112 (emergency phone) for help
Compression: too shallow (%)	Starting CPR
Compression: adequate depth (%)	
Compression: too deep (%)	**Resulting from the Questionnaire**
Compression rate (compression/min)	Correct position of hands
Compression rate: too slow (%)	Correct depth of chest compression
Compression rate: adequate (%)	Correct frequency of chest compressions
Compression rate: too fast (%)	Correct proportion of compressions-ventilations
Ventilation volume (mL)	
Ventilation: too little volume (%)	**Subjective perception (0–10)**
Ventilation: adequate volume (%)	Competence performing CRP
Ventilation: too much volume (%)	Confidence performing CPR in a real OHCA
Hands-off time (s)	
Correct hand position (%)	
30:2 cycles in 2 min (#)	

CPR: cardiopulmonary resuscitation, BLS: basic life support, OHCA: out-hospital cardiac arrest, #: number.

**Table 2:** Descriptive and comparative data between T1 (after the initial training) and T2 (follow-up eight months after the training) for cardiopulmonary resuscitation (CPR) variables.

Variables CPR	T1: Initial		T2: Follow-up		*p* Value ^a^	*t* or *Z ^b^*	Change	
	Mean	(SD)	Mean	(SD)				
Quality of CPR components								
Global CPR performance (%)	72.5	(20.1)	64.3	(29.1)	0.323		↓	11.3%
Compression performance (%)	78.6	(25.9)	65.6	(38.2)	0.168		↓	16.5%
Ventilation performance (%)	69.9	(30.1)	60.1	(35.2)	0.399		↓	14.0%
Compression depth								
Compression depth (mm)	51.4	(7.9)	56.0	(5.7)	0.007	−2.700	↑	8.1%
Too shallow (%)	33.9	(39.1)	16.3	(29.5)	0.010	−2.576	↓	51.9%
Adequate depth (%)	66.1	(39.1)	83.7	(29.5)	0.010	−2.576	↑	21.0%
Too deep (%)	0.0	(0.0)	0.0	(0.0)	1.000			0.0%
Compression rate								
Compression rate (comp/min) ^c^	115.3	(11.5)	101.1	(11.3)	<0.001	5.909	↓	12.3%
Too slow (%)	12.7	(27.0)	49.4	(43.5)	0.001	−3.219	↑	74.2%
Adequate rate (%)	50.4	(40.7)	43.2	(40.5)	0.584		↓	14.3%
Too fast (%)	36.9	(43.5)	7.4	(21.9)	0.001	−3.254	↓	79.8%
Ventilations								
Ventilation volume (ml) ^c^	663.7	(319.0)	657.1	(364.7)	0.934		↓	1.0%
Too little volume (%)	26.0	(31.8)	11.2	(23.6)	0.064		↓	56.9%
Adequate volume (%)	38.2	(30.1)	30.4	(35.3)	0.334		↓	20.5%
Too much volume (%)	35.8	(38.6)	45.4	(41.6)	0.379		↑	21.1%
Others								
Hands-off time (seconds)	7.5	(2.1)	6.0	(1.7)	0.001	−3.216	↓	20.2%
Correct hand position (%)	98.2	(8.8)	77.2	(39.7)	0.028	−2.197	↓	21.3%
30:2 cycles in 2 min (#)	4.5	(1.3)	4.1	(2.2)	0.348		↓	9.6%

Mean values except for percentages. ^a^ Differences between T1 and T2 were assessed with repeated measures *T*-test (or the Wilcoxon test for non-parametric variables). *^b^ t*-value or *Z*-value. ^c^ Variables with a no-normal distribution. #: number.

**Table 3:** Descriptive variables of the CPR components at T1 in participants with CPR performance <70% (*n* = 9).

CPR Variables in Participants with Global CPR Performance Scores <70%	T1: Initial Training	
	Mean	(SD)
Quality of CPR components		
Global CPR performance (%)	52.1	(6.1)
Compression performance (%)	54.7	(9.0)
Ventilation performance (%)	73.3	(11.6)
Compression depth		
Compression depth (mm)	44.7	(2.4)
Too shallow (%)	67.4	(12.6)
Adequate depth (%)	32.6	(12.6)
Too deep (%)	0.0	(0.0)
Compression rate		
Compression rate (compression/min)	115.7	(4.6)
Too slow (%)	13.6	(10.8)
Adequate rate (%)	52.1	(15.8)
Too fast (%)	34.3	(15.8)
Ventilations		
Ventilation volume (mL)	537.0	(95.0)
Too little volume (%)	32.4	(12.6)
Adequate volume (%)	47.9	(11.3)
Too much volume (%)	19.7	(11.2)
Others		
Hands-off time (s)	8.7	(0.8)
Correct hand position (%)	95.3	(4.7)
30:2 cycles in 2 min (#)	4.1	(0.5)

CPR: cardiopulmonary resuscitation. T1: after the initial training. SD: standard deviation. #: number.
